# Prognostic Factors for Cancer-Specific Survival and Disease-Free Interval in 130 Patients with Follicular Thyroid Carcinoma: Single Institution Experience

**DOI:** 10.3390/diagnostics14242817

**Published:** 2024-12-14

**Authors:** Matija Buzejic, Zoran Bukumiric, Branislav Rovcanin, Milan Jovanovic, Marina Stojanovic, Goran Zoric, Katarina Tausanovic, Nikola Slijepcevic, Vladan Zivaljevic

**Affiliations:** 1 Clinic for Endocrine Surgery, University Clinical Center of Serbia, 11000 Belgrade, Serbia; matijabuzejic@gmail.com (M.B.); rovcaninb@yahoo.com (B.R.); milanjovanovicceh@gmail.com (M.J.); goranvanjazoric@gmail.com (G.Z.); katarinatausanovic@gmail.com (K.T.); dr.nikola.slijepcevic@gmail.com (N.S.); 2 Institute of Medical Statistics and Informatics, School of Medicine, University of Belgrade, 11000 Belgrade, Serbia; zoran.bukumiric@med.bg.ac.rs; 3 School of Medicine, University of Belgrade, 11000 Belgrade, Serbia; marinailicstojanovic@gmail.com; 4 Center for Anesthesiology and Resuscitation, University Clinical Center of Serbia, 11000 Belgrade, Serbia

**Keywords:** follicular thyroid cancer, cancer-specific survival, disease-free interval

## Abstract

Background: Follicular thyroid carcinoma (FTC) is categorized into three groups: minimally invasive FTC (MIFTC), encapsulated angioinvasive FTC (EAIFTC), and widely invasive FTC (WIFTC). FTC is the second most common type of thyroid tumor, though it remains relatively rare in the general population. This study aimed to examine the prognosis and prognostic factors in patients with follicular thyroid carcinoma. Methods: Data were obtained from a tertiary referral center for 130 FTC patients, covering the period from 1995 to 2020. Clinical data included demographic characteristics, tumor features, type of surgery, tumor recurrence, and vital status. Descriptive statistical methods, Kaplan–Meier survival curves, and Cox proportional hazard regression were used for statistical analysis to identify independent predictors. Results: Distant metastases occurred in 12 patients during the follow-up period. The 5-year, 10-year, 15-year, and 20-year cancer-specific survival (CSS) rates were 98.1%, 92.3%, 83.5%, and 79.8%, respectively. Independent unfavorable prognostic factors for CSS included widely invasive tumor type (hazard ratio [HR] 3.63, 95% CI 1.29–10.18), multifocality (HR 6.7, 95% CI 1.37–32.72), and presence of distant metastases (HR 2.29, 95% CI 1.08–4.84). When disease-free interval (DFI) was considered, the 5-year, 10-year, 15-year, and 20-year rates were 92.3%, 85.3%, 82.0%, and 76.6%, respectively. Independent unfavorable prognostic factors for DFI were widely invasive tumor type (HR 2.53, 95% CI 1.02–6.28) and tumor multifocality (HR 7.69, 95% CI 1.07–55.17). Conclusions: The 10-year survival rate for patients with FTC is relatively favorable. Factors associated with poorer prognosis include the presence of distant metastases, WIFTC, and multifocality. Factors linked to disease recurrence are WIFTC and multifocality.

## 1. Introduction

Follicular thyroid carcinoma (FTC) is considered as the second most common carcinoma deriving from follicular thyroid cells, and it accounts for approximately 10% of all thyroid malignancies [1,2,3]. Due to its tendency to invade blood vessels, distant metastases are more common than metastases in lymph nodes, which makes FTC more aggressive than papillary thyroid carcinoma (PTC) [4]. FTC occurs more often in areas of iodine deficiency [1,2,3,4,5]. Preoperative diagnosis of FTC is hard to set since follicular adenoma and follicular carcinoma present with very similar cell patterns in fine needle aspiration biopsy. Histologically, in contrast to follicular adenoma, the main characteristic of follicular carcinoma is the presence of capsular and/or vascular invasion [6]. Until 2022, the classification of World Health Organization (WHO) distinguished FTC as minimally invasive follicular carcinoma (MIFTC in which limited capsular and/or vascular invasion is present) and widely invasive follicular carcinoma (WIFTC-with thyroid tissue and/or vascular invasion). The latest WHO classification 5th edition, which was published in 2022, describes three forms of FTC, with adding encapsulated angioinvasive follicular carcinoma as a new entity (EAIFTC) [7,8].

In previously published papers there are contradictions regarding risk factors for FTC [9]. Some of the reasons that could explain these contradictions are the result of a bias present in these studies. In some of the studies the authors included in their study group patients with FTC and patients with oxyphilic tumors (Hurthle cell), which could obviously impact the results. In addition to this study there are only few studies evaluating prognostic factors in patients with “pure” FTC [1,10,11,12,13,14,15,16,17].

The aim of our study was to determine the cancer-specific survival (CSS) and disease-free interval (DFI) in a large series of FTC patients. Additionally, we analyzed prognostic factors and predictors of survival and recurrence of disease in FTC patients.

## 2. Materials and Methods

A retrospective cohort study was conducted, which included 130 patients with FTC who underwent initial surgery between January 1995 and December 2020. Patients with FTC with poorly differentiated carcinoma were excluded from the study. We also excluded all patients with oxyphilic Hurtle cell carcinoma (HTC) (since earlier WHO classifications included HTC as FTC subtype) and patients with inadequate postoperative follow-up (less than 12 months). The study was approved by the ethical committee of our institution. The informed consent was not obtained since the study was retrospective. We collected data from medical documentation of patients (medical history and electronic database at our institution): demographic characteristics (sex, age), length of disease before operation, clinical features (thyroid hormone levels, thyroglobulin (Tg), thyroid peroxidase antibodies, thyrotropin receptors antibodies, preoperative fine needle aspiration biopsy (FNAB), regional/lymph node/distant metastases, infiltration of surrounding organs), histopathological characteristics of tumor (multifocality, tumor diameter, extrathyroidal extension, vascular invasion, capsule invasion); since the 5th edition of WHO classification was published, we revised all pathological findings after which three groups of tumor type was made (according to pathologist report based on vascular and capsular invasion MIFC, WIFC, EAIFTC), type of surgery (total thyroidectomy, lobectomy, total thyroidectomy+ dissection, two stage thyroidectomy), additional treatment forms (radioiodine therapy, substitutional-suppressive thyroxine therapy), additional diseases (diabetes, hypertension (HTA) and other malignant tumors). Since EAIFTCs are classified as a more aggressive type of tumor than MIFTC, we analyzed EAIFTC separately and along with WIFTC in uni- and multivariate analysis [8].

The length of disease was defined as the period from the time of diagnosis of thyroid gland changes until the time of the first surgical procedure. Staging of the tumor was carried out based on TNM classification, which was valid at the time of operation, based on the histopathological findings. Serum levels of thyroid hormones, antibodies, and markers (thyroid-stimulating hormone, triiodothyronine, thyroxine, thyroid peroxidase antibody and thyroglobulin) were measured just before the surgery. Infiltration of the surrounding tissue-extra thyroid extension (ETE) was determined based on intraoperative and histopathological findings. Patients were followed twice per year in the first 5 years after surgery; after the fifth year check-up was once in 12 months. If patients missed regular check-ups, information about health status was obtained through contact with patients (telephone call) or with contact with their family members. We have also collected data from local recurrences and for metastases of tumors in patients with recurrence or metastases and we obtained further treatment methods. Most of the patients who underwent total thyroidectomy received substitutional suppressive levothyroxine therapy. The median follow-up period was 122.45 months (ranging from 15 to 317 months). The disease-free interval (DFI) was calculated as period between the primary treatment of a malignancy and the first sign of tumor recurrence. Cancer-specific survival (CSS) is defined as the duration of time from the first thyroid surgery due to FTC to the death of the patient caused by the cancer, whether it was caused by the original tumor or a second primary tumor of the same cancer type.

### Statistical Analysis

Statistical analysis was conducted using SPSS version 22.0 software (SPSS Inc., Chicago, IL, USA). For time series analyses, the Kaplan–Meier method and log-rank test were used for univariate analysis. Statistical significance was defined as *p* < 0.05. Variables that reached statistical significance in the univariate analysis were subsequently included in the multivariate analysis. Multivariate analysis was performed using logistic regression models, with the assumption of proportionality for explanatory variables verified within the model.

## 3. Results

During the 25-year period at our institution, 17,791 patients were operated due to thyroid gland disease. Among these, thyroid neoplasms were histologically diagnosed in 5546 patients (31.1%). FTC was diagnosed in 130 patients, which represents 2.3% of all thyroid malignances and 0.7% of all patients that were operated on due to thyroid disease.

Basic demographic characteristics of patients with FTC are presented in Table 1. The mean age of patients at the time of surgery was 51.4 ± 14.3 years; the youngest was 18 years old and the oldest one was 82 years. FTC was more common in female patients, with a female to male ratio of 3:1. The mean time from first diagnosis of the thyroid disorder until the time of surgery was 36 months (further defined as length of disease). The mean tumor size was 43.6 mm. Based on WHO criteria, MIFTC was diagnosed in 107 (82.3%) while WIFTC was diagnosed in 23 patients (17.7%). By WHO criteria from 2022, MI, EAI and WI FTC were diagnosed at 103 (79.2%), 6 (4.6%), and 21 (16.2%) respectively. Fine-needle aspiration biopsy was performed on 75 patients (57.7%), most of which were classified as benign (Bethesda II) and suspicion of follicular neoplasm lesion (Bethesda IV), at 33 (44.0%) and 30 (40.0%), respectively.

TNM classification, type of surgery, associated thyroid disorders as well as data about ablative radioiodine therapy are presented in Table 2. Preoperative level of serum thyroglobulin was measured in 43 patients, the median of Tg was 203 ng/L (ranging from 3–1806 ng/L). In three patients (2.3%) distant metastases were diagnosed prior to thyroid surgery, while in 12 patients (9.2%) distant metastases occurred during the follow-up period. Radioiodine therapy was administered to 62 patients (47.3%). At initial surgery, total thyroidectomy was performed in 80 (61.0%) patients, lobectomy in 48 patients (36.6%) after which 7 out of 48 underwent completion of thyroidectomy due to tumor type, diameter of tumor, and age and gender (widely invasive type 2 patients, size of tumor greater than 4 cm 2 patients, age < 45 years and male gender 3 patients). In three patients, total thyroidectomy and central lymph node dissection were performed. During initial surgery in three patients (in whom total thyroidectomy was performed), adjacent musculature was resected due to tumor extension and in one patient, tumor infiltrated the anterior wall of the trachea. In all four of those patients, WIFTC was diagnosed.

Table 3 shows summarized characteristics of patients in whom distant metastases (DMs) occurred during the follow-up period. The female to male ratio was 3:1. The mean age of patients who developed metastases was 51.4 ± 14.3 years. The tumor size was 60 ± 17.4 mm. Among patients with DMs, in five patients MIFTC was diagnosed by the WHO 2016 classification. According to the WHO 2022 classification, MIFTC, EAFTC, and WIFTC were diagnosed in four, one, and seven patients, respectively. The distant metastases were located in the lungs and bones, affecting six patients each. Among the patients with lung metastases, two underwent atypical resection, two underwent segmental resection, and two had only a biopsy performed. In patients with bone metastases, metastasectomy was performed in four cases, while biopsy alone was conducted in two cases. Following histopathological confirmation of distant metastases, all patients received radioiodine therapy.

### 3.1. Disease-Free Interval (DFI)

**Figure 1:**
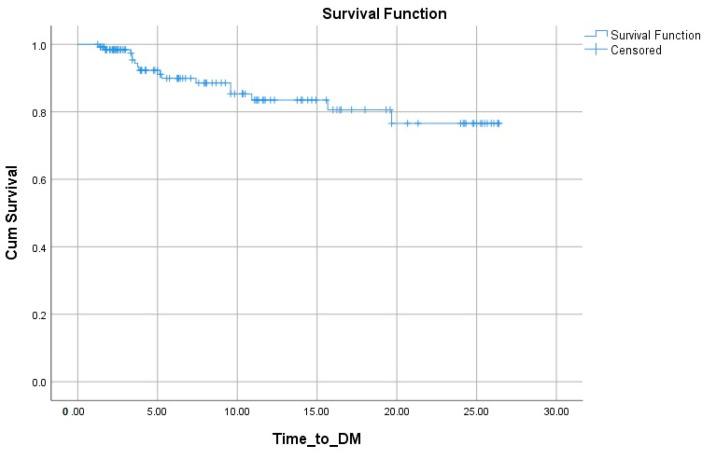
Kaplan–Meier for the disease-free interval of patients with FTC.

The Kaplan–Meier curve for DFI is shown in Figure 1. The estimated mean of DFI was 278.241 months (95% CI 258.033–298.450). The 5, 10, 15, and 20 year DFIs were 92.3%, 85.3%, 82.0%, and 76.6%, respectively.

### 3.2. Cancer-Specific Survival (CSS)

**Figure 2:**
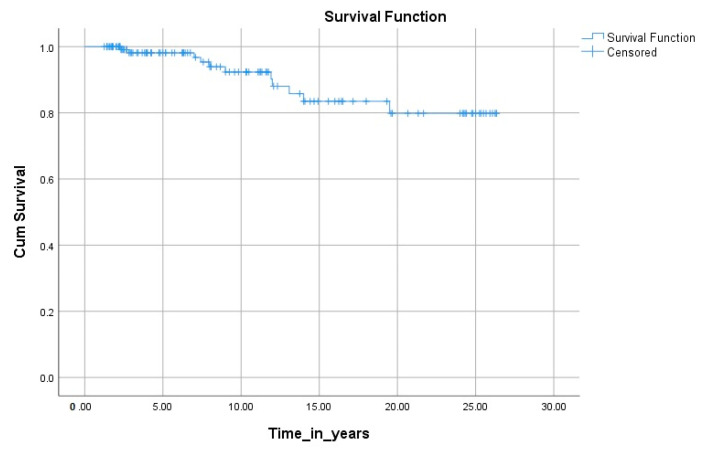
Kaplan–Meier curve for the cancer-specific survival curve of patients with FTC.

Cancer-specific survival (CSS) was 280.899 months (95% CI 261.309–300.488). The 5-, 10-, 15-, and 20-year CSS was 98.1%, 92.3%, 83.5%, and 79.8%, respectively. During the follow-up period, 18 out of 130 patients died (13.8%); 11 died due to disease (8.4%), and 7 out of other causes (5.4%). In Figure 2 cumulative CSS is presented with Kaplan–Meier curve.

In Table 4 are presented summarized results for univariate and multivariate analysis for CSS and DFI.

Univariate analysis for DFI revealed seven factors associated with poorer prognosis: tumor type (EAIFTC and WIFTC), multifocal tumor, tumor size, radioiodine therapy, extra-thyroid extension, and HTA. Multivariate regression analysis showed that the independent unfavorable prognostic factors for DFI were: tumor type (hazard ratio (HR) 2.53 CI 1.02–6.28) and multifocality of the tumor (hazard ratio (HR) 7.69 CI 1.07–55.17). In Table 4 are presented summarized results for univariate and multivariate analysis for DFI.

Univariate analysis for CSS revealed seven factors associated with poorer prognosis: histological type of tumor (EAIFTC and WIFTC), multifocality (one vs. more than one foci of tumor), tumor size (diameter greater than 4 cm), presence of distant metastases, extra-thyroid extension, hypertension, and radioiodine therapy. Multivariate regression analysis showed that the independent unfavorable prognostic factors for CSS were: tumor type (hazard ratio (HR) 3.63 CI 1.29–10.18), multifocality (HR 6.7 CI 1.37–32.72), and presence of distant metastases (DMs) (hazard ratio (HR) 2.29 CI 1.08–4.84). In Table 4 are presented summarized results for univariate and multivariate analysis for CSS.

## 4. Discussion

We conducted this study to examine the prognostic factors for CSS and DFI in patients with FTC. The evaluated factors included age, gender, tumor type and subtype, type of surgery, presence and location of metastatic disease, and concomitant diseases. Factors associated with poorer CSS included: widely invasive tumor type, multifocality, tumor size greater than 4 cm, presence of distant metastases, extrathyroidal extension, hypertension, and radioiodine therapy. Independent unfavorable prognostic factors for CSS were tumor type, multifocality, and the presence of distant metastases, indicating that patients with the widely invasive tumor type, multiple tumor foci, or distant metastases had poorer survival rates. For DFI, the unfavorable prognostic factors were tumor type and tumor multifocality, meaning that patients with widely invasive type and tumor with more than one foci are at greater risk for developing DMs or recurrence of disease. Although hypertension was identified as a factor associated with poorer outcomes for both CSS and DFI, it was not determined to be an independent unfavorable prognostic factor in multivariate analysis. Hypertension is one of the most common concomitant diseases in the general population. Furthermore, studies have established that patients with hypertension, particularly males over the age of 40, tend to exhibit more invasive features of papillary thyroid cancer. For this reason, we included the most frequent concomitant diseases in our analysis [18]. After reviewing the literature, we identified numerous studies investigating prognostic factors for survival in patients with FTC. It is worth noting that the term “Hürthle cell carcinoma” has been replaced by “neoplastic cell carcinoma” in the fifth edition of the WHO Classification of Thyroid Tumors [8]. Most of the studies included patients with not only “pure” FTC but also the oxyphilic type of FTC. As our study focused solely on “pure” FTC, we excluded these mixed studies from our review and found only nine studies specifically addressing “pure” FTC. Table 5 summarizes the studies and their patient demographic characteristics.

The mean age of patients in our study was 51.4 years, which aligns with findings from other studies. Lin et al. and Yamazaki et al. reported average ages of 56.3 and 51 years, respectively, in their cohorts. In contrast, studies by Chow et al., Lo et al., Lee et al., Su et al., and Badulescu et al. reported slightly lower mean ages of 49, 44, 41.5, 46.6, and 48.8 years, respectively [1,10,11,12,13,14,15,16,17]. In our study, age >45 years was not a significant factor for poorer prognosis. Similarly, Lin et al. and Chow et al. did not find a correlation between age and poorer prognosis. However, Yamazaki et al. reported that patients older than 55 years had a poorer prognosis, while Su et al. found that age >60 years increased the cause-specific hazard of FTC-related death. In the study by Lo et al., poorer prognosis was correlated with age >40 years for men and >50 years for women [1,10,11,12,13,14,15,16,17].

Previously, Stenson et al. reported that male gender is predictor for poorer prognosis, the explanation for this finding could be that thyroid carcinomas could be detected in advanced stage in male than in female patients [19]. In our study, multivariate analysis did not show that male sex was an independent predictor of poorer both CSS and DFI.

The mean tumor size in our study was 4.3 cm, but in group of patients who developed DMs mean size was 6 cm, which is a bit larger than the sizes reported by Su et al., Badulescu et al., and Chow et al., at 3 cm, 3.8 cm, and 3.6 cm, respectively [1,11,16]. In the study by Kim et al., no significant difference was observed in the size of follicular adenomas compared to follicular carcinomas [20]. In our univariate analysis for CSS, a tumor size >4 cm was associated with a worse outcome, but there was no significance in multivariate analysis.

Multivariate regression analysis in our study for CSS showed that the presence of DMs, multifocality and WIFTC were unfavorable prognostic factors. During the follow-up period, 18 out of 130 patients died (13.8%), with 11 deaths attributed to the disease (8.4%) and 7 to other causes (5.4%). Out of 11 deaths due to disease, in 3 patients DMs occurred prior to surgery WIFTC was diagnosed, 7 patients died due disease in whom DMs were revealed during follow-up (WIFTC-5, EAI-1, MIFTC-1), and 1 patient with DMs had local recurrence with the infiltration of trachea as cause of death (WIFTC type). A total of nine patients diagnosed with WIFTC died due to the disease. The percentage of reported deaths in other studies ranged from 0% in the studies of Podda et al. and Badalescu et al., up to 24.1% reported by Shen et al. [13,15,17].

In this cohort, DMs were identified in three patients (2%) prior to thyroid surgery. During the follow-up period, DMs occurred in 12 patients (9.2%), resulting in a total of 15 patients (11.5%) with DMs. The most common sites of metastases were the bones and lungs, a finding consistent with other studies. For instance, Lo et al. reported that 13.4% of patients had DMs, whereas Podda et al. observed a significantly lower DM occurrence rate of 4%; all cases were diagnosed with WI FTC [12,13]. Shen et al. reported a high DMs occurrence rate of 30%, with 21% of patients having DMs before surgery and 9% developing it during the follow-up period [16]. In our study, multivariate regression analysis revealed that multifocality and WIFTC were independent unfavorable prognostic factors for poorer DFI. The estimated mean DFI in our cohort was 278.241 months. Wu et al. observed that 15.3% of patients with FTC had DMs prior to surgery, and 7.4% developed DMs during the follow-up period. Across all studies, including ours, the most common sites for distant metastases were the lungs and bones [21]. Additionally, DMs were more frequently observed in patients with WIFTC compared to those with MIFTC, consistently reported across all studies [1,10,11,12,13,14,15,16,17].

Lymph node metastases are rare in FTC, since vascular spread is more common. Some studies have reported cases of lymph node metastases, identifying it as a prognostic factor for poorer survival. Su et al. found that lymph node metastases significantly affected only the cumulative incidence of FTC mortality [1]. In our study, only one patient presented with ipsilateral lymph node metastases. While most studies have shown a neutral effect of lymph node metastases on survival rates, some have suggested that it predicts a worse prognosis. A limitation of these studies is that they included patients with the oxyphilic variant of FTC, which, according to earlier classifications, is more likely to involve lymph node metastases than non-oxyphilic FTC [21,22,23]. In a study by Ito et al., the prognosis for non-oxyphilic FTC was worse than that of oxyphilic FTC, as demonstrated by both univariate and multivariate analyses [4].

In univariate analysis, radioiodine therapy was a risk factor for poorer prognosis. It can be explained by the fact that at our clinic all patients with WIFTC, tumor size larger than 3 cm and multifocal FTC are sent to RI. Also, most of the patients after total thyroidectomy received RI. All patients with DMs received another dose of RI immediately after DMs was surgically removed (when it was feasible).

Ito et al. published several studies regarding the number of foci of vascular invasion as one of the major factors for poorer prognostic factors. They subdivided vascular invasion (VI) positive cases into two categories (namely, VI(1+) and VI(2+)) according to the number of invasion sites [4]. Distant recurrence free survival was EAIFTC/VI(1+) tended to be poorer than that of WIFTC/VI(–) suggesting that the degree of VI significantly affects patients’ prognoses in EAIFTC cases [4,16].

Geographical variations in the incidence of FTC may be attributed to differences in dietary iodine content. In iodine-deficient areas, the relative rate of FTC is typically higher, reaching up to 40% of differentiated thyroid carcinoma cases in some regions [22]. Since preoperative diagnosis of FTC is challenging, most patients diagnosed with FTC undergo hemithyroidectomy rather than total thyroidectomy [24].

FTC is primarily a unifocal disease, and lobectomy is usually the preferred surgical approach for MIFTC. However, when EAIFTC or WIFTC is diagnosed—especially in the presence of nodules in the contralateral lobe—completion thyroidectomy is recommended [25]. In our study, there was no significant difference in prognosis between total thyroidectomy and lobectomy.

Fine-needle aspiration biopsy was performed on 75 patients (57.7%) as a standard preoperative diagnostic method. Most biopsies were classified as benign (Bethesda II) or suspicious for follicular neoplasm (Bethesda IV) in 33 (44.0%) and 30 (40.0%) cases, respectively. Despite 44% of biopsies being benign, surgery was performed due to the size of the nodules, confirming that preoperative FNAB is not entirely reliable for diagnosing FTC. This limitation arises because vascular and capsular invasion—key diagnostic features of FTC—cannot be assessed preoperatively [24].

Limitations of our study are like in previous studies. The large time frame of the sample included in our study may influence the therapeutical goals due to improvement of technology through the years. The absence of DMs in patients included in the study does not necessarily mean that distant metastases did not occur (it can be hypothesized that some distant metastases were not detected due to avoidance of imaging examinations or due to lack of physical signs). Also, some confounding factors, such as patient comorbidities (in our study most common comorbidities were included in statistical analysis such as HTA and diabetes mellitus) and relevant molecular markers (e.g., BRAF, RAS, TERT, etc.), could have had an impact on the results.

## 5. Conclusions

Widely invasive type of tumor, multifocality, and the presence of distant metastases are associated with poorer cancer-specific survival in FTC patients. A shorter disease-free interval is more common in patients with widely invasive type of tumor or multifocality.

Patients with FTC who underwent total thyroidectomy can benefit from RAI. This simplifies the follow-up of these patients and aids in detecting recurrence of disease. Further studies and meta-analyses should be performed including only studies regarding FTC.

## Figures and Tables

**Table 1:** Baseline characteristics and WHO classification of patients with follicular thyroid carcinoma.

Variables	*n* = 130
Age, mean ± sd (years)	51.4 ± 14.3
Age (years)	
<45	39 (30%)
45	91 (70%)
Sex, *n* (%)	
male	32 (24.6%)
female	98 (75.4%)
Lenght of disease, median (range)	36 (1–240)
WHO 4th edition *n* (%)	
Minimally invasive	107 (82.3%)
Widely invasive	23 (17.7%)
WHO 5th edition *n* (%)	
Minimally invasive	103 (79.2%)
Encapsulated angioinvasive	6 (4.6%)
Widely invasive	21 (16.2%)
FNAB	
Bethesda I	4 (3%)
Bethesda II	34 (26.1%)
Bethesda IV	30 (23%)
Bethesda V	4 (3%)
Associated thyroid disease	
Micropapillary carcinoma	9 (6.9%)
Thyroiditis	13 (10%)
Graves’ disease	1 (0.7%)
Concomitant diseases	
Diabetes mellitus yes	12 (8.2%)
No	118 (90.8%)
Arterial hypertension yes	48 (37.2%)
No	81 (62.8%)

FNAB—fine needle aspiration biopsy.

**Table 2:** TNM, Initial surgical procedure, surgeon experience, associated thyroid disease, and radioiodine therapy following surgery.

Variables	*n* = 130
T	
T1	9 (6.9%)
T2	65 (50%)
T3	47 (36.1%)
T4	9 (6.9%)
*n*	
N0	129 (99.3%)
N1b	1 (0.7%)
M	
M0	115 (88.5%)
M1	15 (11.5%)
Tg serum level ng/L, median (range)	203 (3–1806)
Type of surgery	
Total thyroidectomy	70 (53.8%)
Lobectomy	51 (39.2%)
Total thyroidectomy + dissection	2 (1.7%)
Two stage thyroidectomy	7 (5.3%)
Radioiodine therapy	
Yes	62 (47.6%)
No	68 (52.4%)

Tg—thyroglobulin; M0—no metastases, M1—metastases prior and after thyroid surgery.

**Table 3:** Characteristics of patients who developed distant metastases during follow-up period.

Variables	*n* = 12 (M2)	*n* = 3 (M1)
Age, mean ± sd (years)	57.2 ± 14.6	58.3 ± 8.9
Age (years)		
<45	10 (83.3%)	1 (33.2%)
45	2 (16.7%)	2 (66.8%)
Gender		
Male	3 (25%)	1 (33.2%)
Female	9 (75%)	2 (66.8%)
WHO 2016		
Mi	5 (41.7%)	2 (66.8%)
Wi	7 (58.3%)	1 (33.2%)
WHO 2022 MI EAI WI	4 (33.4%) 1 (8.3%) 7 (58.3%)	2 (66.8%) 1 (33.2%)
Tumor size-mean ± sd-mm	60 ± 17.4	43.3 ± 5.7
Localization of distant metastases		
Lung	6 (50%)	2 (66.8%)
Bone	6 (50%)	1 (33.2%)
Time to distant metastases (months)	99.3	

M2—metastases occurred during follow up; M1—metastases present prior to thyroid surgery.

**Table 4:** Univariate and multivariate analysis for CSS and DFI.

	Univariate			Multivariate		
	p	HR	95%CI	p	HR	95%CI
CSS						
Tumor type (widely vs. minimally invasive)	0.001	3.94	1.98–7.85	0.014	3.63	1.29–10.18
Tumor type						
Minimally invasive						
Encapsulated angioinvasive	0.003	20.53	2.87–146.66	0.060	12.83	0.89–183.47
Widely invasive	0.001	19.24	3.97–93.15	0.040	9.76	1.10–85.98
Multifocal tumor (yes vs. no)	0.001	8.91	2.70–29.34	0.019	6.71	1.37–32.72
Distant metastases + (M2 vs. metastasis free patients)	0.001	3.61	1.91–6.85	0.029	2.29	1.08–4.84
Tumor size (<4 cm vs. >4 cm)	0.042	3.98	1.05–15.05	0.606	1.55	0.29–8.33
Radioiodine therapy (yes vs. no)	0.033	9.41	1.23–73.58	0.911	0.86	0.06–11.30
Extrathyroid extension (yes vs. no)	0.001	2.00	2.37–25.56	0.508	0.52	0.07–3.58
Age (<45 years vs. >45 years)	0.120	51.53	0.36–7.38			
Gender (male vs. female)	0.641	1.44	0.31–6.67			
Type of surgery (total+ vs. less than total thyroidectomy)	0.127	0.30	0.06–1.40			
Bethesda system	0.348	0.78	0.48–1.29			
Tg serum level (<500 vs. more than 500 (ng/L))	0.759	0.99	0.98–1.00			
Diabetes mellitus (yes vs. no)	0.755	1.38	0.17–10.89			
HTA (yes vs. no)	0.003	7.84	2.05–29.93			
Length of disease (<5 vs. >5 years)	0.717	0.80	0.24–2.63			
DFI						
Tumor type (widely vs. minimally invasive)	0.001	3.47	1.89–6.36	0.045	2.53	1.02–6.28
Tumor type						
Minimally invasive						
Encapsulated angioinvasive	0.039	5.32	1.09–25.98	0.053	5.59	0.97–31.95
Widely invasive	0.001	6.33	2.20–18.19	0.380	2.08	0.40–10.71
Multifocal tumor (yes vs. no)	0.09	5.79	1.55–21.69	0.042	7.69	1.07–55.17
Tumor size (<4 cm vs. >4 cm)	0.008	7.71	1.68–35.3	0.094	4.45	0.77–25.70
Radioiodine therapy (yes vs. no)	0.017	12.00	1.54–93.25	0.253	4.23	0.35–50.45
Extrathyroid extension (yes vs. no)	0.001	8.33	2.63–26.38	0.749	0.76	0.14–4.07
Age (<45 years vs. 45 years)	0.085	3.83	0.83–17.75			
Gender (male vs. female)	0.973	0.97	0.26–3.62			
Type of surgery (total+ vs. less then total thyroidectomy)	0.527	0.67	0.20–2.25			
Bethesda system	0.782	1.05	0.72–1.56			
Tg serum level (<500 vs. more than 500 ng/L)	0.724	1.00	0.99–1.00			
Diabetes mellitus (yes vs. no)	0.189	2.78	0.60–12.78			
HTA (yes vs. no)	0.031	3.61	1.12–11.67			
Length of disease (<5 vs. >5 years)	0.842	1.13	0.33–3.87			

Tg—thyroglobulin; HTA-hypertension; CSS—cancer-specific survival; DFI—disease-free interval; HR—hazard ratio; CI—confidence interval.

**Table 5:** Published papers regarding pure FTC.

Author (Year)	No. of Patients	No. (%) of Death Due Disease	No. (%) of Patients with Recurrent Disease or DMs	Female/Male	Mean Age
Lin (1999) [10]	205	25 (12.2)	69 (33.6)	53/152	56.3
Chow (2002) [11]	215	37 (17.2)	32 (14.8)	159/56	49
Lo (2005) [12]	156	17 (10.8)	17 (10.8)	131/25	44
Podda (2015) [13]	71	0	7 (9.8)	62/9	
Lee (2017) [14]	166	4 (2.4)			41.5
Su (2019) [1]	204	30 (14.7)	34 (16.6)	155/49	46.6
Badalescu (2020) [15]	133	0	16(12)	114/19	47.8
Yamazaki (2021) [16]	478	10(2)	20 (4.1)	356/122	51
Shen (2023) [17]	153	37 (24.1)	46(30)	99/54	54.3
Our study	130	11 (8.4)	12 (9.2)	98/32	51.4

DMs—distant metastases.

## Data Availability

The original contributions presented in the study are included in the article, further inquiries can be directed to the corresponding author.
